# Supplementary material for: MetaRibo-Seq measures translation in microbiomes
Source: Nat Commun. 2020 Jun 29;11:3268. doi: 10.1038/s41467-020-17081-z (PMC7324362; doi:10.1038/s41467-020-17081-z)
Supplement: Supplementary file 10 — Supplementary Data 7 [file 41467_2020_17081_MOESM10_ESM.zip › File2/Confidence_VeryHigh_Taxonomy/368623_out.krona.html]

Javascript must be enabled to view this page.

members
magnitude
magnitudeUnassigned
count
unassigned
taxon
rank

368623\_out

7

superkingdom
2
7

phylum
976
7

class
7
200643

7
171549
order

family
171552
3

genus
838
3


SRS893324\_contig\_number\_15528
species
28131
1

28136
1

SRS013836\_contig\_number\_contig-100\_19.78738
species


SRS148361\_contig\_number\_1033
species
1
470565


SRS023352\_contig\_number\_41124SRS1054881\_contig\_number\_20459SRS144512\_contig\_number\_49339SRS893291\_contig\_number\_6587
species
1497953
4
